# Our Visitors

**Published:** 1905-10

**Authors:** Henry K. Leake

**Affiliations:** Chairman of Arrangement Committee


### Correspondence.

### Our Visitors.

THE JOURNAL has received the following from the committee on arrangements:

*Texas Medical Journal:*

According to definite arrangement, Dr. Joseph Price will be in Dallas on October 30th and 31st next. There is a strong probability that he will be accompanied by Dr. John Deaver of Philadelphia and Dr. McMurtry of Louisville. The two last named gentlemen now have the matter under consideration and will make an earnest effort to be present here with Dr. Price and take an active part in a meeting with the profession of Texas that promises to be enjoyable and instructive.

Papers will be read and operations performed by the distinguished visitors.

It is planned by the profession of Dallas to have every feature of this meeting on a scale commensurate with the professional standing of our visitors and it is hoped that physicians throughout the State will co-operate with us to impress them with that hospitality and scientific merit that is characteristic of the medical profession of our great State. Consequently the meeting will be more State than local in its import and such is the view that is taken of it by the eminent surgeons themselves; certainly the Texas medical profession will not disappoint their expectations.

In due time a well arranged program will be published in the Dallas morning *News* and other newspapers.

HENRY K. LEAKE, M. D.
Chairman of Arrangement Committee.

